# Effects of Online Cooperative Learning on Students’ Problem-Solving Ability and Learning Satisfaction

**DOI:** 10.3389/fpsyg.2022.817968

**Published:** 2022-06-10

**Authors:** Yi-Ping Wang, Tung-Ju Wu

**Affiliations:** ^1^College of International Relations, Huaqiao University, Xiamen, China; ^2^School of Management, Harbin Institute of Technology (HIT), Harbin, China

**Keywords:** information technology, online cooperative learning, problem-solving ability, learning satisfaction, social skills

## Abstract

As technology changes, it is becoming more common in education for students to acquire knowledge from sources other than just their teachers. In the face of a diverse student background, teachers have to make adjustments in their instruction so that students do not simply listen. Student-based educational philosophy aims to combine instructional methods with cooperative learning to allow students to change from passive learning to active knowledge construction, reinduce students’ learning motivation and passion, and enhance students’ self-learning effectiveness. Focusing on college students in Fujian Province as the research sample, 360 copies of a questionnaire were distributed for this study. After deducting invalid and incomplete ones, 298 copies remained, with a retrieval rate 83%. The research results showed significantly positive correlations between online cooperative learning and problem-solving ability, problem-solving ability and learning satisfaction, and online cooperative learning and learning satisfaction. According to the results, it is expected, in the digital era, to integrate information technology into the teaching environment and focus on learning objectives to create teaching software with a user-friendly interface, simple operation, learning process recording, and an interactive learning community in the teaching-learning process to develop the characteristics and effectiveness of digital teaching and learning.

## Introduction

As times progress and technology improves, teachers are no longer the only channel for students acquiring knowledge. Students in this generation are stimulated by distinct and diverse cultures to show more active and flexible characters or responses than students before them, and are even brave enough to challenge existing values. Students in a traditional learning model with passive lectures will not concentrate in the classroom. Examinations have been a core part of education for a long time. It is the best time to practice cooperative learning. The curricula show that the ideas such as taking the initiative, engaging in the public, and seeking the common good are important. Engaging in the public and seeking the common good is a result of the characters of positive independence and face-to-face fostering of interactive and interpersonal skills mentioned in cooperative learning. In this respect, it can be stated that cooperative learning guides students to be well and develops various interactive abilities with ego, others, society, and nature. It also helps students in applying and practicing their knowledge, experiencing the meaning of life, being willing to devote to the sustainable development of society, nature, and culture, and seeking reciprocity of each other and common good. Information technologies are material tools that learners should actively and broadly apply to a the positive interaction channel between oneself and the environment to effectively engage the public with others and the environment (Li et al., 2021).

In the face of diverse student background, teachers have to make adjustments in their instruction to stop students from simply listening. Educational philosophy should be student-based to promote each student’s thinking. In this case, cooperative learning allows students to change from passive learning into active knowledge construction, could reinduce students’ learning motivation and passion, and enhance students’ self-learning effectiveness. Most students are digital natives born after 1980, while most of their teachers are digital immigrants and even “digital refugees” escaping from technologies and being afraid of new knowledge. The overlap between such two generations is limited, meaning that their values and morality are distinct. Modern students are digital natives able to use mobile phones, televisions, computers, laptops, and tablets since childhood, and highly dependent on new technologies. Information-technology-integrated instruction with multimedia equipment and materials means teaching and learning is no longer restricted to dictation and paper-and-pencil (Vaz et al., 2021); the class climate has changed to cooperative learning. The operation of cooperative learning is smoother through information technology, and a communication and interaction bridge can be built through information technology so that cooperative learning could cultivate students’ problem-solving ability to further promote learning satisfaction. As a result, the effects of online cooperative learning on students’ problem-solving ability and learning satisfaction are discussed in this study, expecting to integrate information technology into the teaching environment in the digital era, focus on learning objectives based on learning theory, have teaching software with a user-friendly interface, simple operation, learning process recording, and an interactive learning community in the teaching-learning process to develop the characteristics and effectiveness of digital teaching and learning.

## Literature Review and Hypothesis

Constructivists regard gaining knowledge as a comprehensive and reflective thinking activity through students’ independent exploration and observation and highly praise learner-centered learning environments. Teachers’ roles of propagating the doctrine, imparting professional knowledge, and resolving doubts change into knowledge building facilitators. The superordinate-subordinate relationship of “Learning from Teacher” is changed into the equal relationship of “Learning with Teacher.” The learning perspective of constructivism facilitates the development of current learning technology (Cortez et al., 2021).

Dozens of instructional strategies are developed for cooperative learning, and each grouping method presents the characteristics and applicable teaching situation. Teachers could flexibly apply the difference according to instructional objectives, student characteristics, and course attributes. Researchers, in the interview with collaborative teachers, also reveal not being restricted into a grouping method, but extracting the advantages of various methods, and making flexible adjustments in consideration of teachers’ personality traits and class attributes and characteristics (Akdemir et al., 2020). Major cooperative learning strategies are classified into three types, including one suitable for leading sharing and discussion among students, another for assisting students in mastering learning content, and the last for leading teams for theme-based inquiry. Each type shows various strategies to cope with different teaching styles, or more than two strategies could be changed and applied depending on the demands (Hafeez, 2021).

Li and Keller (2018) mentioned the significant effects of using web problem-based cooperative learning and on the problem-solving skills of the children. The results revealed the better performance of students compared to traditional problem-based learning. Del Gaudio et al. (2021) used online cooperative learning to discover the advantages and strengths, solve problems according to collaborative interaction, comprehend the roles, integrate the discussed ideas, clearly master the tasks, coordinate the allocation of team members’ reports, complete reports according to previous discussion results, discuss and modify successive measures together, inspect cooperation results, track back problem-solving processes, and reflect team organization and roles, problem-solving ability as to independently complete tasks with high-level thinking, and cooperative problem-solving ability as to create the value of synergy, solve problems and complete tasks together, and create good performance beyond the expectation (Wu et al., 2019, 2022). Ingrid (2019) explained that independent thinking and analysis ability allowed dealing with daily life and even life problems. Teachers applying information technology to cooperative learning to enrich students’ life experience, being good at asking questions, creating problem-solving teaching situations, applying technological tools to speculate and deduce problems, effectively solving problems with cooperative discussions, and enhancing adaptability to life could help students become problem-solving experts. For this reason, the following hypothesis is established in this study.

*H1*: Online cooperative learning presents significantly positive correlations with problem-solving ability.

*H1-1*: Online cooperative learning shows significantly positive correlations with problem-solving ability.

*H1-2*: Online cooperative learning reveals remarkably negative correlations with problems-solving ability.

Oates and Ritók (2018) explained that learners being able to effectively enhance their problem-solving ability after going through the curriculum arranged by the school, course content of teachers, and effective promotion of knowledge acquisition in the learning process, with consistent expectation and anticipation, would appear satisfactory; on the contrary, dissatisfaction would be delivered. Metin-Orta and Demirtepe-Saygılı (2021) stated that education aimed to help individuals live their life; in real situations, an individual using critical thinking to solve complicated and messy dilemmas and problems was the core task of modern education. Teachers in the teaching process did not simply transmit knowledge, provide guidance for study, and dispel confusion, but had to help students associate old experience with new knowledge to further solve problems through tight cognition structure to form meaningful learning in order to effectively enhance learning satisfaction. Wu et al. (2021) regarded cooperative problem-solving ability as an individual with sufficient ability communicating and dialoging with more than two companions to share knowledge and skills, collaboratively and effectively participate in an activity, and develop teamwork ability to solve problems. Collaborative problem solving referred to several partners collaboratively completing a task where each partner had to positively participate (Chiao and MacVaugh, 2021; Min et al., 2021), mutually coordinate, and pull together to solve problems in the task with teamwork so as to effectively enhance learning satisfaction. Accordingly, the following hypothesis is establishment in this study.

*H2*: Problem-solving ability shows remarkably positive correlations with learning satisfaction.

*H2-1*: Problem-solving ability appears to have notably positive correlations with learning satisfaction.

*H2-2*: Problem-solving ability presents significantly negative correlations with learning satisfaction.

Wu et al. (2020) applied interactive APP to analyze learning satisfaction with idiom teaching; the students, regardless of gender and learning achievement, were satisfied with the use of interactive APP for idiom learning. The use of information-technology-integrated cooperative learning for the learning achievement of students in the experimental group did not outperform students in the control group, but the learning satisfaction was better than those in the control group. Kurilovas and Kubilinskiene (2020) mentioned that students in the experimental group with cooperative learning outperformed students with general cooperative learning on learning achievement and learning attitude and presented positive learning satisfaction. Haidar and Fang (2019) explained cooperative learning as teachers effectively applying information technology to smooth cooperative learning; for instance, dynamic information materials and real-time team performance could assist in students’ learning motivation, learning ambition, learning satisfaction, and learning effectiveness and create a quality learning environment with peer teamwork and teacher-student interaction. The following hypothesis is therefore established in this study.

*H3*: online cooperative learning reveals notably positive correlations with learning satisfaction.

*H3-1*: Online cooperative learning shows remarkably positive correlations with learning satisfaction.

*H3-2*: Online cooperative learning reveals notably negative correlations with learning satisfaction.

## Methodology

### Operational Definition

#### Online Cooperative Learning

Online cooperative learning, as the independent variable in this study, is measured with positive interdependence, promotive interaction, social skills, and group processing, according to the blended learning model proposed by Liao et al. (2019).

1. Positive interdependence: mutual dependence, mutual responsibility, mutual help, acceptance of assistance, and cheering up team members.
2. Promotive interaction: mutual assistance, sharing information, and providing clear explanation in the team.
3. Social skills: leadership and communication.
4. Group processing: evaluating the cooperation effectiveness of each other.

#### Problem-Solving Ability

Problem-solving ability, as the dependent variable in this study, is measured with exploration and comprehension, planning and execution, and monitoring and reflection, according to the problem-solving ability model proposed by Lin et al. (2018).

#### Learning Satisfaction

Learning satisfaction, as the dependent variable in this study, is measured with student aspects, teacher aspects, and school aspect, according to the blended learning model proposed by Travis and Bunde (2020).

1. Student aspects: including students’ interests, learning motivation, learning attitude, personality traits, gender, needs, experience, learning ability, learning effectiveness, and peer interpersonal relationship.
2. Teacher aspects: covering teachers’ professional ability, traits, teaching methods, curriculum arrangement, teaching content, difficulty in material design, attitude towards students, and teacher-student interaction model.
3. School aspects: containing school equipment, learning environment, environmental safety and health, teaching resources, and transportation.

### Research Object and Analysis Method

College students in Fujian Province, as the research sample, were distributed 360 copies of a questionnaire for this study. After deducting invalid and incomplete ones, 298 copies were valid, with a retrieval rate 83%. After confirming the applicable online cooperative learning strategy, the actual teaching activity is practiced as planned. Four teachers practicing cooperative learning in the school were invited as the collaborative teachers to deliver the 10-week (total 50 sessions) teaching activity to 500 students in 10 classes of a university in Fujian Province. The questionnaire data collection is preceded after the end of the course.

Two-stage analysis in Structural Equation Modeling (SEM) is applied to analyze goodness-of-fit and test the model in this study. Confirmatory Factor Analysis (CFA) is first used, aiming to test the existence of independent variables in the model in order to delete dependent variables with bad effects on causal analysis. Path analysis is then preceded after the modification. Path analysis aims to estimate the relationship of model paths among variables. Without Confirmatory Factor Analysis to test independent variables, the use of path analysis might be affected by independent variables to result in bad goodness-of-fit or insignificant model paths. Goodness-of-fit test in Amos18.0 is utilized in this study. CMIN/DF of the measurement result being smaller than 5 is acceptable and being smaller than 3 is excellent; GFI, AGFI, NFI, IFI, TLI, and CFI are better higher than 0.9; and RMR, RMSEA, and SRMR are better when smaller and ideally smaller than 0.05.

## Results

### Factor Analysis

The online cooperative learning scale in this study, with factor analysis, extracted four factors of “positive interdependence” (eigenvalue = 2.633, *α* = 0.84), “promotive interaction” (eigenvalue = 1.875, *α* = 0.86), “social skills” (eigenvalue = 2.236, *α* = 0.81), and “group processing” (eigenvalue = 1.633, *α* = 0.87). The cumulative covariance explained achieves 75.923%. The problem-solving ability scale, after factor analysis, extracted three factors of “exploration and comprehension” (eigenvalue = 3.251, *α* = 0.86), “planning and execution” (eigenvalue = 2.407, *α* = 0.88), and “monitoring and reflection” (eigenvalue = 2.716, *α* = 0.83). The cumulative covariance explained reaches 77.493%. The learning satisfaction scale, with factor analysis, extracted three factors of “student aspects” (eigenvalue = 1.577, *α* = 0.80), “teacher aspects” (eigenvalue = 2.281, *α* = 0.85), and “school aspects” (eigenvalue = 2.388, *α* = 0.90). The cumulative covariance explained achieves 80.762%.

### Empirical Analysis Model of Structural Equation

Regarding the Confirmatory Factor Analysis (CFA) results, the convergent validity of the observation model could observe the reliability of individual observed variable, construct reliability (CR), and average variance extracted (AVE); the reliability of individual observed variable is better higher than 0.5. The factor loadings of observed items in this study are higher than the suggested value. The construct reliability is better higher than 0.6, while other researchers suggest higher than 0.5 being acceptable. The model calibration results reveal the construct reliability higher than 0.5. Average variance extracted is suggested higher than 0.5; the average variance extracted of the dimensions in this study is higher than 0.5, conforming to the suggested value.

**Figure 1:**
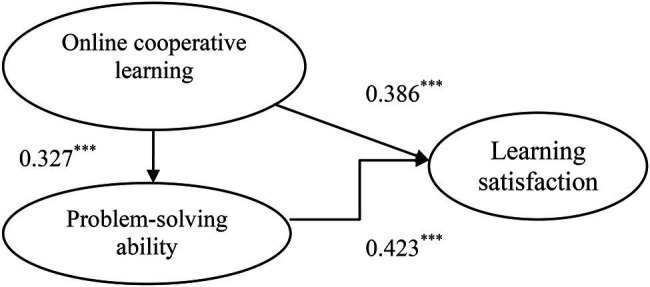
Model path diagram. *** *p* < 0.001.

In terms of the structural formula calibration results, *χ*^2^/*df*, RMSEA, GFI, AGFI, RMR, and NFI are suggested to be ≦5, ≦0.08, ≧0.9, ≧0.9, ≦0.05, and ≧0.9, respectively. This study shows *χ*^2^/*df* = 3.142≦5, RMSEA = 0.032≦0.08, GFI = 0.967≧0.9, AGFI = 0.934≧0.9, RMR = 0.031≦0.05, and NFI = 0.918≧0.9, revealing good overall model fit. Under good overall model fit, the structural formula parameter calibration results are shown in Table 1 and Figure 1. The research results present online cooperative learning → problem-solving ability 0.327^***^ that H1 is supported, problem-solving ability → learning satisfaction 0.423^***^ that H2 is supported, and online cooperative learning → learning satisfaction 0.386^***^ that H3 is supported.

**Table 1:** Structural equation modeling result.

Parameter/evaluation standard	Coefficient
Online cooperative learning → problem-solving ability	0.327^***^
Problem-solving ability → learning satisfaction	0.423^***^
Online cooperative learning → learning satisfaction	0.386^***^
*χ*^2^/Degree of Freedom ≦ 5	3.142
Root Mean Square Error of Approximation (RMSEA) ≦ 0.08	0.032
Goodness-of-Fit Index (GFI) ≧ 0.9	0.967
Adjusted Goodness-of-Fit Index (AGFI) ≧ 0.9	0.934
Root Mean Square Residual (RMR) ≦ 0.05	0.031
Normed Fit Index (NFI) ≧ 0.9	0.918

*** p < 0.001.

## Discussion

The research results prove that, in the practice of online cooperative learning, information technology makes up for the insufficiency of cooperative learning, enriches courses, promotes students’ learning motivation, and drives learning effectiveness to form a positive cycle. Students’ learning motivation comes from the advancement of performance and the learning confidence comes from the ideal performance. Teachers use online cooperative learning to facilitate group discussion skills and the understanding of students. They also use Google Forms to conduct digitalized tests, and mind maps and tables to improve students’ problem-solving skills (Simamora, 2017). In the teaching-learning process, instructional objectives are inspected to return the teaching profession. Teachers are good at asking questions to enhance students’ cooperation and encourage thinking. Especially in comprehension and analysis, the top-down relationship should be broken and the subjective consideration of teachers’ cognition, ideas, and interpretation as being better than students should be avoided so that it would not come out with teachers’ expected answers (Phillips et al., 2014). Students’ answers could be typed with computers to respect the answers, enhance the confidence without losing students’ creativity, and present brainstorming; teachers ensure the focus and integration at the end. The application of online cooperative learning could reconstruct teachers’ teaching profession, and the experience and constant rolling correction could improve teaching skills to face changeable students and present the value of online cooperative learning. The intervention of information technology could change the resistance to the online cooperative learning process into assistance, helping it to become a powerful backup force of online cooperative learning, induce learning motivation, and promote problem-solving ability and learning satisfaction as the final instructional objectives.

Alves et al. (2019) explained collaborative problem solving as an individual or more than two companions with sufficient capability sharing knowledge and skills through communication and dialogue, collaboratively and effectively participating in activities, and developing teamwork to solve encountered problems. Collaborative problem solving referred to a task being collaboratively completed by several partners. Each partner had to positively participate, mutually coordinate, and help each other in the same situation to solve problems with teamwork so as to effectively enhance learning satisfaction. The intervention of information technology could make the best out of a bad situation in the online cooperative learning process to support online cooperative learning, induce learning motivation, and promote problem solving capability and learning satisfaction as the ultimate instructional objectives. The research result conforms to the points of view proposed by Munawar and Chaudhary (2019) and Haidar and Fang (2019).

Teachers need full training to guide students with “stretching and jumping” opportunities in the “interactive relationship.” Meanwhile, teachers need full wisdom to help students move from conflict compromise to positive trust (Ramdani et al., 2019). What is more, multiple evaluations outside the classroom, such as completion of team assignments, quiz performances, and sectional examination performance, help teams not to slack. Besides, each member is important that no-one is confident of the winning (Hafeez, 2021). Students would search network data, discuss grounded arguments, focus on discussion through information technology, and save a lot of time for groupwork. Teachers, with statistics, would announce team performance with data at any time to induce competition and crisis awareness of teams. There might be conflict in a team, but a contest with multiple evaluations allows individuals to give up personal prejudice and unite to make effort for the team. It naturally reinforces the group process of cooperative learning (Akdemir et al., 2020).

## Conclusion

The research results show that the item of “*Teachers currently use the instructional method of online cooperative learning to make courses interesting and active*” receives the highest score in online cooperative learning strategies, revealing the acquisition of student identity. The item of “*I think the use of platform[s] for Internet communication media could help the communication and teamwork between team members and I in the cooperative learning course*” receives the highest score in problem solving capability, revealing the acquisition of student identity. The item of “*I think the application of online cooperative learning could enhance learning ability and confidence*” receives the highest score in learning satisfaction, revealing the acquisition of student identity.

The research results prove that students’ responses in class are a mirror reminding teachers of the need to adjust the instructional methods. In traditional didactic instruction, students’ academic achievement decides teachers’ success. In the use of online cooperative learning, students’ learning motivation awakes teachers’ passion. Teachers could continuously retain the original instructional methods; nevertheless, modern students are active and there are special students who are extroverts or introverts. These students may challenge teachers’ authority. Teachers can easily get tired if they do not adapt their instructional methods according to the diverse needs of students. The assistance of information technology in the practice allows seeking consensus from online resources in the team discussion. Under the situation with a well-grounded argument, students are convinced by each other to contribute to the successive discussions. The research result conforms to the points of view proposed by Weaver et al. (2019) and Ingrid (2019). With online cooperative learning, teachers simply combine the original computer software with cooperative learning courses through the Internet, rather than re-learning brand new and strange computer software. Teachers who enjoy learning and self-growth could challenge themselves and activate teaching with advanced functions. However, it should be kept in mind that information technologies are only tools; using media can attract students’ attention in a short period, but having students internalize knowledge is the goal. Karakus Taysi (2019) mentioned the aims of education as helping individuals live their life. The development of individual critical thinking and problem-solving skills are the main aims of contemporary education. Teachers did not simply propagate the doctrine, impart professional knowledge, and resolve doubts in the teaching process, but had to help students link old experience with new knowledge, make tight cognitive structures for meaningful learning, and further solve problems to effectively promote learning satisfaction.

Online cooperation learning method is important for cultivating students’ independent thinking, interpersonal communication, competition awareness, and teamwork (Cortez et al., 2021). Teachers and students are good at utilizing information technology to have students focus on discussion content and direction, instantaneously acquire the answers and feedback and correction, and improve team performance with data (Mutua and Ong'ong'a, 2020). When making effort in the learning process, the learning result would not be lower than the expected performance and students would reflect this with their learning satisfaction.

## Data Availability Statement

The original contributions presented in the study are included in the article/Supplementary Material, further inquiries can be directed to the corresponding author.

## Ethics Statement

This study was reviewed and approved by the ethics committee of the Huaqiao University. Written informed consent was obtained from all participants for their participation in this study.

## Author Contributions

Y-PW performed the initial analyses and wrote the manuscript. T-JW assisted in the data collection and data analysis. All authors revised and approved the submitted version of the manuscript.

## Funding

This research was supported by the National Natural Science Foundation of China (71702059).

## Conflict of Interest

The authors declare that the research was conducted in the absence of any commercial or financial relationships that could be construed as potential conflicts of interest.

## Publisher’s Note

All claims expressed in this article are solely those of the authors and do not necessarily represent those of their affiliated organizations, or those of the publisher, the editors and the reviewers. Any product that may be evaluated in this article, or claim that may be made by its manufacturer, is not guaranteed or endorsed by the publisher.
